# Nonvital Pulp Therapy in Primary Teeth Using Mineral Trioxide Aggregate Obturation: A Retrospective Case Series

**DOI:** 10.1155/crid/8096584

**Published:** 2026-04-01

**Authors:** Tetsuhide Makiguchi, Yoshi Terauchi, Kingsley Wong, George Bogen

**Affiliations:** ^1^ Department of Pediatric Dentistry, Tokyo Dental College, Chiyoda City, Japan, tdc.ac.jp; ^2^ Department of Endodontics, Tokyo Medical and Dental University, Bunkyo City, Japan, tmd.ac.jp; ^3^ Telethon Kids Institute, The University of Western Australia, Perth, Western Australia, Australia, uwa.edu.au; ^4^ Department of Endodontics, University of Queensland, School of Dentistry, Brisbane, Queensland, Australia, uq.edu.au

**Keywords:** case series, exfoliation, MTA cement, primary teeth resorption, pulpectomy, succedaneous teeth

## Abstract

Nonvital pulp therapy is a treatment option that can preserve pathologically affected primary teeth and contribute to space maintenance. This retrospective case series examined the healing effects in infected primary teeth after pulpectomy (PE) using mineral trioxide aggregate (MTA) obturation and eruption characteristics of the succedaneous teeth. Twenty‐one teeth in 17 patients (mean age: 7 years 1 month) were selected for PE using MTA and diagnosed with pulp necrosis, periapical/furcation radiolucencies (67%, *n* = 14), resorptive defects (62%, *n* = 13), failing previous PEs (33%, *n* = 7), pulpotomies (29%, *n* = 6), or histories of trauma. All accessible canals were obturated with MTA and restored teeth monitored up until the time of natural exfoliation or planned extraction. Teeth with and without pretreatment root resorptive defects were evaluated over a 6‐year period for MTA extrusion, posttreatment healing, preservation periods, residual MTA retention, radiographic connection between the tooth lesion and follicle, succedaneous tooth eruption times, and enamel defects. Posttreatment evaluations revealed healing or stabilization of supporting tissues without signs of recurrent infection in all cases. All primary teeth exfoliated or were extracted successfully and replaced by unaffected permanent teeth. Residual MTA cement was not detected in supporting bone after exfoliation. In cases where residual MTA was detected in soft tissues after exfoliation, the cement was shed during permanent tooth eruption in all cases. In five cases, MTA was curetted and removed along with partially resorbed roots at the time of extraction or after exfoliation (23.8%, *n* = 5). The average preservation period was 2 year 3 months (SD 1 year 3 months). The replacement period for contralateral primary teeth was not significantly different (paired *t*‐test *p* value = 0.49). This retrospective case series suggests that MTA obturation/extrusion can promote healing and prolonged retention of infected primary teeth without affecting normal successor tooth eruption or newly formed enamel.

## 1. Introduction

Preserving primary teeth until they naturally exfoliate is important for maintaining arch space integrity, normal masticatory function, and eruption guidance for permanent teeth [1, 2]. Pediatric patients with advanced caries or trauma in the mixed dentition often require definitive treatments such as vital pulp therapy (VPT) and non‐VPTs including pulpectomy (PE)^1^ in primary teeth, root canal therapy in permanent teeth (RCT), apexification, apexogenesis, or regenerative procedures [1–3]. However, infected primary teeth diagnosed with pulp necrosis and apical or furcation pathosis are frequently extracted instead of treated, even though they could benefit from PE [4]. This is often due to anatomical features, technical difficulties, presence of extensive pathoses, age and health history, parent/patient expectations, access to care barriers for children, and patient management considerations [1, 2, 5].

In cases where PE and root canal obturation are elected, traditional filling materials include calcium hydroxide (CH), zinc oxide–eugenol (ZOE) cement, iodoform (IOD) pastes, and various ZOE, CH, or IOD‐based combination products [2, 6–8]. Some of these filling materials have shown acceptable outcomes when used for canal obturation [2, 9, 10]. However, these materials can have drawbacks and can cause resorption, enamel hypoplasia, and ectopic eruption of successor teeth [9, 11]. At present, there is no clear agreement on the best obturation material for primary teeth [6, 8, 11–13].

Mineral trioxide aggregate and other calcium silicate–based cements (CSCs) have shown excellent results in permanent teeth and are increasingly used in pediatric dentistry [14–16]. CSC application to treat primary and immature permanent teeth in children has shown favorable results in teeth diagnosed with pulp necrosis and open apices, internal and external root resorption, apical periodontitis, and reversible/irreversible pulpitis when used for VPT [17–21]. Bioactive endodontic cements demonstrate excellent sealing ability and strong biocompatibility, promote hard tissue repair, and exhibit superior antimicrobial properties attributed to sustained high pH and intratubular mineralization [16, 20, 22, 23].

Considering the physicochemical properties of CSCs, bioceramic cements like MTA could be suitable root canal filling materials when used for primary teeth exhibiting advanced resorption, open apices, or failing previously treated PEs [16, 18, 19, 22–26]. These features of CSCs may provide advantages over conventional PE agents that are contraindicated for use in primary teeth exhibiting advanced pathological resorption [1, 2, 9, 21]. Interestingly, MTA obturation in primary teeth has only been described in cases with congenitally missing successor teeth [27, 28].

**Figure 1:**
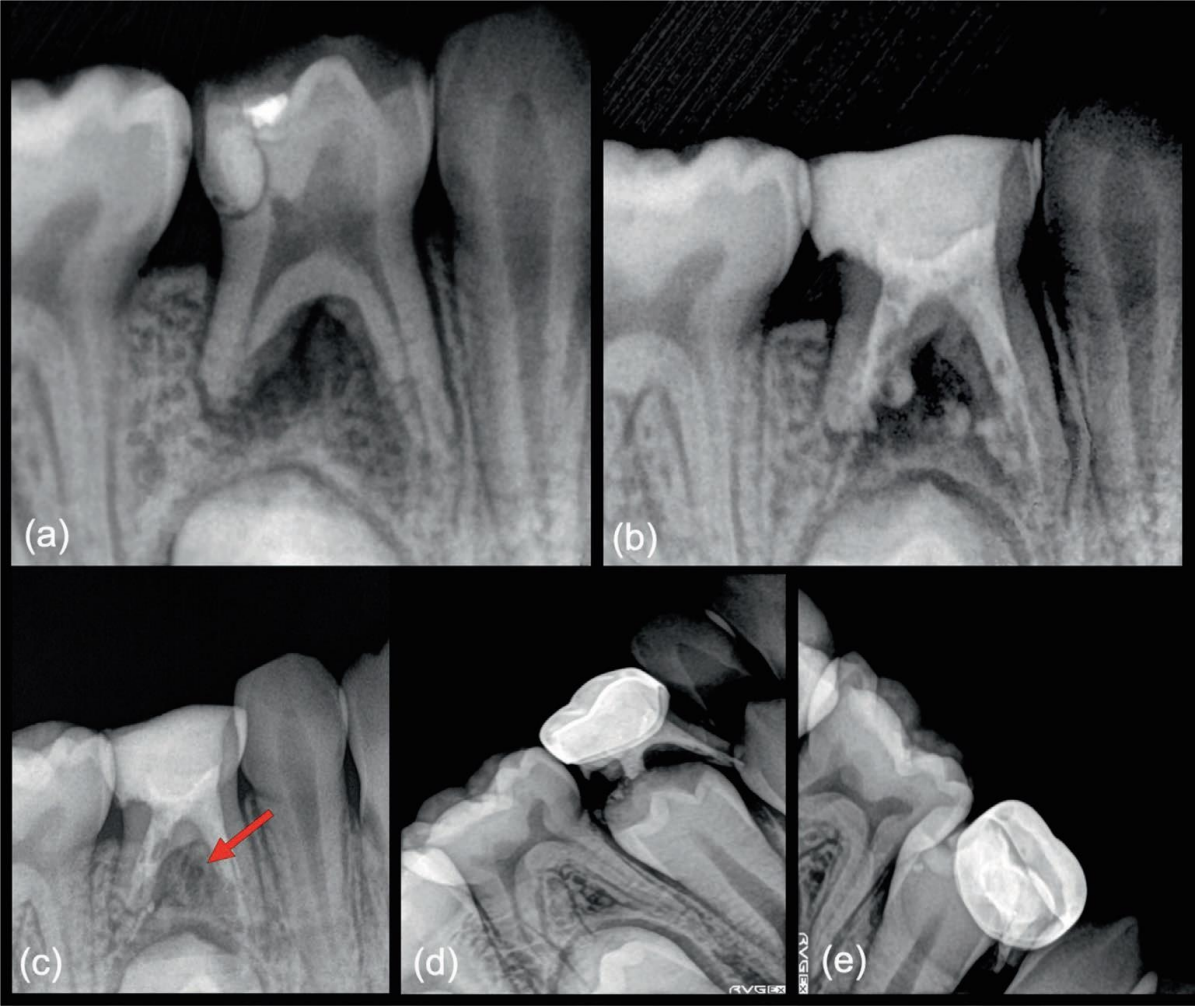
Initial pulpectomy case treated by MTA obturation (Case 1). (a) Preoperative periapical radiograph of a mandibular right first primary molar (No. S) showing a disto‐occlusal composite with recurrent caries. The tooth displays a combined furcation and apical lesion, with pathologic root resorption evident on the distal and mesial roots. The patient had swelling, a sinus tract, and was diagnosed with pulp necrosis. (b) Post‐MTA obturation image showing MTA extrusion in the furcation from the medial walls of both roots and apex of the distal root. (c) Posttreatment radiograph review at 2 months, showing advanced remineralization of the combined furcation/apical lesion (arrow). The patient was asymptomatic. (d) Two‐year 3‐month recall radiograph showing normal exfoliative‐related resorption. (e) Three‐year, 2‐month radiograph prior to elective extraction.

Despite promising case reports, long‐term studies on the use of MTA and other CSC materials for obturation in primary teeth are limited. This retrospective case series began after a young patient presented with a symptomatic first primary molar displaying a sinus tract and failed composite restoration. The tooth was diagnosed with pulp necrosis. Radiographically, the molar revealed a combined apical/furcation radiolucency and was diagnosed with symptomatic apical periodontitis associated with external root resorption

(Figure 1). The primary molar was treated with PE, including chemomechanical preparation and MTA obturation. Resolution of symptomology and the sinus tract with advanced osseous remineralization of supporting bone was observed after 2 months. After a uneventful extraction to promote routine eruption, the successor tooth eruption time showed no significant difference compared to the contralateral tooth with no residual MTA detected in the supporting bone.

Based on the favorable outcome of this initial case, an additional 20 subsequent treatment cases were collected and reported here. The MTA obturated primary teeth were monitored until the time of exfoliation or extraction and eruption of the successor teeth. The objective of the retrospective case study report were to evaluate the healing capacity and performance of PE using MTA obturation, examine effects of cement extrusion, assess cement biodegradation after exfoliation/extraction, and identify developmental crown defects/hypoplasia and eruption patterns of the succedaneous teeth.

## 2. Materials and Methods

### 2.1. *Study Design and Patient Inclusion*

This retrospective cases series study was conducted in line with the Preferred Reporting Of CasE Series in Surgery (PROCESS) guidelines for reported findings [29]. In this retrospective case series outcome‐based sampling, 21 cases from 17 patients were followed until the time of successor tooth eruption. The 21 cases included 13 with pretreatment root resorptive defects and eight without root defects. Cases were selected from 130 PEs performed in a private pediatric practice in Tokyo, Japan, over a 6‐year period between 2015 and 2021. Ninety of the 130 patients received MTA obturation and 40 patients were treated with Vitapex (Neo Dental International, Tacoma WA). Seventy‐three of the 90 MTA/PE‐treated patients were lost to follow‐up (81%). This was partly due to the COVID‐19 pandemic and the children in Japan receiving all dental care pro bono. There were no complications or adverse events recorded for any treatments. The patients selected either visited the clinic regularly and could be easily monitored, were referred after unsuccessful treatment by another clinic, or diagnosed for extraction but the parents wished to save the tooth. Medical histories revealed no contraindications to treatment or extraordinary patient management concerns.

Pretreatment clinical assessment established the restorability of teeth without excessive mobility (≤ Grade 2). Teeth were required to have at least 2 mm of sound supra‐gingival dentin surrounding the cervical area and at least three remaining coronal walls. Patients diagnosed with pulp necrosis exhibited various symptoms, including gingival swelling or sinus tracts. Patients treated also presented with previously treated and failing PEs, pulpotomy, or histories of trauma. Imaging revealed one or several conditions, including advanced radicular and/or apical resorption greater than 1 mm [9], periapical/furcation pathosis, and associations between the tooth and follicles of the succedaneous teeth. Teeth exhibiting natural root resorption greater than 2/3 of root length and close to exfoliation or pathological resorption exceeding 50% of the remaining root structure were excluded.

Parents or guardians of patients were given information regarding risks, benefits, and outcomes prior to treatment. The potential for postexfoliation retention of residual MTA cement requiring intervention was explained and acknowledged. No patients treated in this study required antibiotics or received oral, intravenous, inhalation, or general anesthesia/sedation.

### 2.2. Clinical Procedure

All treatments were completed by single, primary, pediatric dental specialist with over 40 years of clinical experience (TM). After guardian approval and written consent, patients received local infiltration or block anesthesia using Lidocaine 0.5% (Nissin Pharmaceutical Co. Ltd., Tendo‐City, Japan) and dental dam isolation for all treatments. All treatments were completed using 3.0X magnification loupes (Carl Zeiss Co. Ltd., Oberkochen, Germany), in one or two visits. Teeth that were treated in two visits received CH intracanal dressing (Ultradent, South Jordan, UT) and were provisionalized with Copr‐Seal Cement Pink (GC Corp., Tokyo, Japan). After caries excavation, canals were accessed and irrigated with 10 mL 6% sodium hypochlorite solution. Working lengths were confirmed using K‐files (MANI, Inc., Tochigi, Japan) and periapical radiographs without the use of electronic apex locators that can be inaccurate in cases with open apices and resorptive defects [30]. All accessible canals were prepared to Master Apical File (MAF) size No. 20–30 with K‐files using 6% sodium hypochlorite/saline irrigation and dried with paper points. Because MTA marginal adaptation is unaffected by smear layer removal, final irrigation with ethylenediaminetetraacetic acid (EDTA) is unnecessary for MTA obturation [23]. MTA was prepared according to the manufacturer′s instructions or mixed to a lower viscosity using sterile water when indicted for obturating narrower canals (ProRoot MTA, Dentsply Tulsa Dental Specialties, Johnson City, TN). The mixture was placed over canal orifices and compacted with smaller than MAF K‐files using a counterclockwise turn and push technique. The procedure was repeated and augmented with ultrasonic activation of the K‐file (Lawaty method) [15]. All canals were filled 0.5 mm short of the apex. Radiographs were taken during the obturation to confirm compaction density in the apical 3–4 mm.

In some cases where molar roots had notable curvature, nickel–titanium No. 10‐25/30 0.04 taper rotary file (ProTaper Gold, Densply/Sirona, Charlotte, NC) instrumentation was used for canal preparations. Ascending MTA compaction beginning 0.5 mm from the radiographic apex was completed using one to two sizes smaller than MAF nickel–titanium files (ProTaper) placed in reverse mode (Auger technique) [31]. All pulp chamber floors were covered with a minimum of 2‐mm MTA thickness to enclose possible accessory canals at the furcation area. The teeth were sealed with either glass ionomer cement (GCFuji IILC, Fuji Dental K.K., Tokyo, Japan) or composite core build‐up (Beautiful Kids Resin, SHOFU Inc., Kyoto, Japan) and restored with prefabricated crowns. Anterior teeth were restored with strip crowns (3 M Pedoform, 3 M ESPE, St. Paul, MN) and molars either with stainless steel crowns (3 M ESPE, St. Paul, MN) or zirconia crowns (NuSmile, Houston, TX) cemented with BioCem Universal (Nusmile, Houston, TX). Definitive restorations were placed at the same appointment or within 7–10 days after completed obturation to avoid recontamination and potential microleakage.

After exfoliation or at time of elective extraction, periapical radiographs were taken to assess the presence of any remaining roots or residual MTA. In cases where MTA was not eliminated after extraction or exfoliation (retained roots), residual MTA was gently removed using light curettage combined with soft tissue water spray irrigation/flushing. In cases recalled after exfoliation showing residual MTA associated with partially resorbed or nonresorbed roots, the area received local anesthetic infiltration. Roots were removed along with intact MTA using serrated forceps No. F300 or F301 (Hu‐Friedy, Chicago, IL, United States). Removal of residual MTA particles in soft tissues was augmented by Miller Surgical Curette No. CM8/CM9 (Hu‐Friedy, Chicago, IL, United States) along with Curette L Cvd. spoon tipped tissue pliers No. 11324 (YDM Corp, Tokyo, Japan) combined with water irrigation/flushing. Complete postoperative instructions were provided to the parents or guardians in each case.

### 2.3. Clinical and Radiographic Assessments

All clinical observations of swelling, sinus tracts, suppuration, tooth restorability, and mobility were assessed and recorded before treatment initiation. Periapical radiographs were evaluated for restorability, resorption, furcation, and periapical radiolucencies. Follow‐up visits that evaluated soft tissue healing, mobility, succedaneous tooth eruption pattern, and enamel malformations or hypomineralization were recorded 6–20 months after exfoliation or extraction. All observations and assessments were completed by the primary operator.

Radiographic preoperative and postoperative assessments were performed independently by two endodontists (YT and GB). Calibration was performed prior to case assessment using radiographs from 12 random PE cases not included in the main study sample. To minimize bias during the case series assessment, radiographs were anonymized and presented in a random order for independent review, followed by the calculation of interrater reliability. Each case was evaluated for internal and external resorption, apical and furcation lesions, and MTA extrusion. This also included the radiographic relationship between the primary tooth apices and the successor tooth follicle and evidence of post‐treatment healing or stabilization. Interexaminer agreement was measured using Cohen’s kappa coefficient (resorption: 0.60; furcation lesions: 0.67; extrusion: 0.59; and connection between primary tooth and follicle: 0.70). Any disagreements between the reviewers were resolved through discussion to reach a final consensus assessment for each case.

### 2.4. Outcome Criteria

Posttreatment outcomes for PE treatments were determined by examining the extent of clinical and radiographic healing, exfoliation time intervals between the involved and contralateral tooth, retention of extruded MTA, and eruption patterns and enamel irregularities of the succedaneous teeth. The prescribed outcome guidelines were modified from criteria established by Coll and Sadian [9].

*Clinical* criteria for acceptable outcomes at 6–12 months included the following:

• Absence of posttreatment sinus tract, suppuration or localized swelling.
• Normal mobility, excluding exfoliative resorption‐associated mobility.
• Absence of symptomology/discomfort at follow‐up reviews.

*Radiographic* criteria included the following:

• Absence or cessation of preexisting root resorption.
• Osseous remineralization or stabilization of preoperative furcation lesions.
• Periapical healing without re‐emergence of apical disease.

### 2.5. Statistical Analysis

Descriptive statistics, including frequencies, percentages, means, and standard deviations/ranges, were used to summarize patient demographics, clinical and radiographic characteristics, and treatment outcomes. The retention/replacement period of the primary tooth, defined as the time elapsed between the date of treatment and the date of natural exfoliation or extraction, was calculated and summarized descriptively. Fisher′s exact test was used to compare proportions between groups. To compare the eruption timing of the permanent successor tooth relative to the contralateral tooth, a paired *t*‐test was employed based on the calculated time difference. Interrater reliability for the radiographic assessments was quantified using Cohen′s kappa coefficient. A *p* value of less than 0.05 was considered statistically significant for all inferential statistical tests. Statistical analysis was performed using Stata Statistical Software (Release 18, StataCorp LLC, College Station, TX).

## 3. Results

A total of 21 cases in 17 patients were treated with follow‐up periods ranging from 11 months to 5 years and 10 months. The first case of MTA primary tooth obturation that initiated this case series was followed for 3 years and 2 months (July 2015 to September 2018). After an uneventful elective extraction along with the removal of residual MTA enclosed within the accompanying soft tissue was confirmed in 2017, 20 additional cases (from 16 individuals) were evaluated. The 17‐patient cohort included nine males and eight females. The mean age at treatment of the 21 cases was 7 years and 1 month (SD 1 year and 11 months), ranging from 1 year and 10 months to 10 years and 2 months. Most treated teeth were molars (86%, *n* = 18), and the remainder maxillary central incisors (14%, *n* = 3) (Table 1).

**Table 1:** Patient demographics and pre‐treatment characteristics in 21 pulpectomy cases of primary teeth using mineral trioxide aggregate obturation.

Case	Pt ID	Sex	Tooth #	Tooth type	Pretreatment characteristics						
							Unsuccessful previous treatment				
					Swelling	Sinus tract/suppuration	Restoration failure/recurrent caries	Pulpectomy	Pulpotomy	Direct pulp capping	Trauma
1	1	M	S	First molar	Yes	Yes	Yes	No	No	No	Yes
2	2	M	L	First molar	Yes	No	No	No	No	Yes	No
3	3	F	T	Second molar	Yes	No	No	Yes	No	No	No
4	3	F	L	First molar	Yes	No	No	No	Yes	No	No
5	3	F	S	First molar	Yes	No	No	Yes	No	No	No
6	4	M	S	First molar	Yes	No	No	Yes	No	No	No
7	5	F	L	First molar	Yes	Yes	No	No	Yes	No	No
8	6	F	B	First molar	Yes	Yes	Yes	No	No	No	No
9	6	F	I	First molar	Yes	No	Yes	No	No	No	No
10	7	M	E	Central incisor	No	No	No	No	No	No	Yes
11	8	M	L	First molar	Yes	No	Yes	No	No	No	No
12	9	F	L	First molar	Yes	Yes	No	No	Yes	No	No
13	10	M	B	First molar	Yes	No	Yes	Yes	No	No	No
14	11	M	S	First molar	Yes	No	No	No	Yes	No	No
15	12	M	L	First molar	Yes	No	No	No	Yes	No	No
16	13	F	L	First molar	Yes	No	No	No	Yes	No	No
17	14	F	F	Central incisor	Yes	Yes	No	Yes	No	No	Yes
18	15	F	K	Second molar	Yes	Yes	Yes	No	No	No	No
19	16	F	B	First molar	No	Yes	No	No	No	Yes	No
20	17	M	F	Central incisor	No	No	No	Yes	No	No	Yes
21	10	M	T	Second molar	Yes	No	No	Yes	No	No	No

More than half of the teeth were retreatments of previous PEs or pulpotomies (62%, *n* = 13). Regarding pretreatment clinical characteristics, the majority of cases presented with swelling (86%, *n* = 18). Sinus tracts and/or suppuration were evident in one‐third of the patients (33%, *n* = 7). Most of the teeth were unsuccessful previous treatments (90%, *n* = 19), including failing restorations or recurrent carries (28.5%, *n* = 6), PEs (33%, *n* = 7), and pulpotomies (28.5%, *n* = 6). Two cases were failed direct pulp caps (14%) with subsequent infection and swelling (*n* = 2) and malposition of a successor tooth (*n* = 1). Three cases (14%) were traumatized teeth, with two resulting in internal resorption or an exposed pulp.

Radiologically, the majority exhibited periapical/furcation radiolucencies (67%, *n* = 14). Additionally, nearly two‐thirds (62%, *n* = 13) had resorptive defects, and approximately three‐quarters (76%, *n* = 16) showed radiographic connections between the tooth lesion and the follicle of the permanent tooth. Evidence of MTA extrusion was observed in 81% of cases (*n* = 17) in the posttreatment radiographs after obturation. Two authors independently verified these radiographic assessments to ensure agreement (YT and GB). The interexaminer agreement of the four features was considered moderate to substantial (0.59–0.70) [32]. The retention periods ranged from 8 months to 5 years and 9 months, with an average preservation period of 2 years and 2 months (SD 1 year and 3 months). Close to one‐third (29%, *n* = 6) of teeth underwent normal exfoliation while 71% (*n* = 15) were electively extracted. Extractions were indicated to prevent ectopic or delayed eruptions. In five cases, MTA was curetted and removed along with partially resorbed roots at the time of extraction or after exfoliation (23.8%, *n* = 5). This occurred in 3/6 (50%) of exfoliated teeth compared to 2/15 (13%) of extracted teeth. This difference was not statistically significant (Fisher′s exact test, *p* = 0.115).

Posttreatment radiographs and clinical examinations showed no signs of infection, inflammation, or recurrent disease. The treated primary teeth exfoliated or were extracted successfully and replaced by permanent teeth without residual MTA cement in the bone or soft tissues. The replacement period for the corresponding primary teeth on the contralateral side (mean 2 years and 9 months [SD 1 year and 6 months]) was significantly longer (approximately 7 months) than that of the treated teeth (paired *t*‐test, *p* = 0.017). Treatments performed on the contralateral primary teeth included resin filling (43%, *n* = 9), PE (29%, *n* = 6), and pulpotomy (14%, *n* = 3) (Table 2).

**Table 2:** Pre‐ and posttreatment characteristics in 21 pulpectomy cases of primary teeth using mineral trioxide aggregate obturation.

Case	Diagnosis	Retreatment	Resorptive defects	Furcation/periapical radiolucency	MTA extrusion	Radiographic connection	Age at treatment	Age at exfoliation	Follow‐up time	Exfoliation status	Residual MTAcuretted	Follow‐up time of contralateral tooth	Treatment of contralateral tooth
1	NP/SAP	No	Yes	Yes	Yes	No	7 years 10 months	11 years 0 months	3 years 2 months	Extracted	No	3 years 6 months	Resin filling
2	NP/SAP	No	No	Yes	No	Yes	8 years 6 months	9 years 5 months	0 years 11 months	Extracted	No	0 years 7 months	Resin filling
3	PT	Yes	No	Yes	Yes	Yes	7 years 8 months	9 years 2 months	1 years 5 months	Natural Shedding	Yes	5 years 1 months	Pulpotomy
4	PT	Yes	Yes	Yes	Yes	Yes	7 years 9 months	9 years 2 months	1 years 5 months	Natural shedding	No	1 years 3 months	Root canal filling
5	PT	Yes	Yes	Yes	Yes	Yes	7 years 11 months	9 years 0 months	1 years 1 months	Natural shedding	Yes	1 years 3 months	Root canal filling
6	PT	No	Yes	Yes	Yes	Yes	6 years 2 months	8 years 7 months	2 years 4 months	Natural Shedding	Yes	4 years 7 months	Resin filling
7	PT	No	Yes	Yes	Yes	Yes	8 years 3 months	10 years 8 months	2 years 5 months	Extracted	No	2 years 9 months	Root canal filling
8	NP/SAP	No	No	No	Yes	Yes	8 years 11 months	10 years 5 months	1 years 6 months	Extracted	No	1 years 6 months	Root canal filling
9	NP/SAP	No	Yes	No	Yes	Yes	9 years 0 months	10 years 3 months	1 years 2 months	Extracted	No	1 years 10 months	Root canal filling
10	NP/SAP	No	Yes	No	No	No	3 years 8 months	7 years 1 months	3 years 4 months	Extracted	Yes	2 years 11 months	No treatment
11	NP/SAP	Yes	No	Yes	No	Yes	10 years 2 months	11 years 10 months	1 years 7 months	Extracted	No	1 years 11 months	Pulpotomy
12	PT	No	Yes	Yes	Yes	No	6 years 5 months	8 years 2 months	1 years 9 months	Extracted	No	2 years 3 months	Resin filling
13	PT	Yes	No	No	Yes	Yes	7 years 5 months	8 years 9 months	1 years 3 months	Extracted	No	2 years 4 months	Resin filling
14	PT	No	Yes	Yes	No	No	5 years 10 months	11 years 2 months	5 years 3 months	Extracted	No	5 years 5 months	Resin filling
15	PT	Yes	Yes	Yes	Yes	Yes	5 years 9 months	7 years 3 months	1 years 6 months	Extracted	Yes	3 years 4 months	Resin filling
16	PT	No	Yes	Yes	Yes	Yes	5 years 6 months	7 years 8 months	2 years 2 months	Extracted	No	0 years 10 months	Root canal filling
17	PT	Yes	Yes	No	Yes	Yes	5 years 10 months	6 years 7 months	0 years 8 months	Extracted	No	1 years 4 months	No treatment
18	NP/SAP	No	Yes	Yes	Yes	Yes	9 years 2 months	11 years 3 months	2 years 1 months	Natural shedding	No	3 years 2 months	Resin filling
19	NP/SAP	Yes	No	Yes	Yes	Yes	8 years 2 months	10 years 2 months	2 years 0 months	Extracted	No	2 years 7 months	Resin filling
20	PT	Yes	No	No	Yes	Yes	1 years 10 months	7 years 8 months	5 years 10 months	Natural shedding	No	6 years 5 months	No treatment
21	PT	No	No	No	Yes	No	7 years 5 months	10 years 2 months	2 years 9 months	Extracted	No	3 years 1 months	Pulpotomy

Abbreviations: NP, necrotic pulp; PT, previously treated; SAP, symptomatic apical periodontitis.

## 4. Discussion

**Figure 2:**
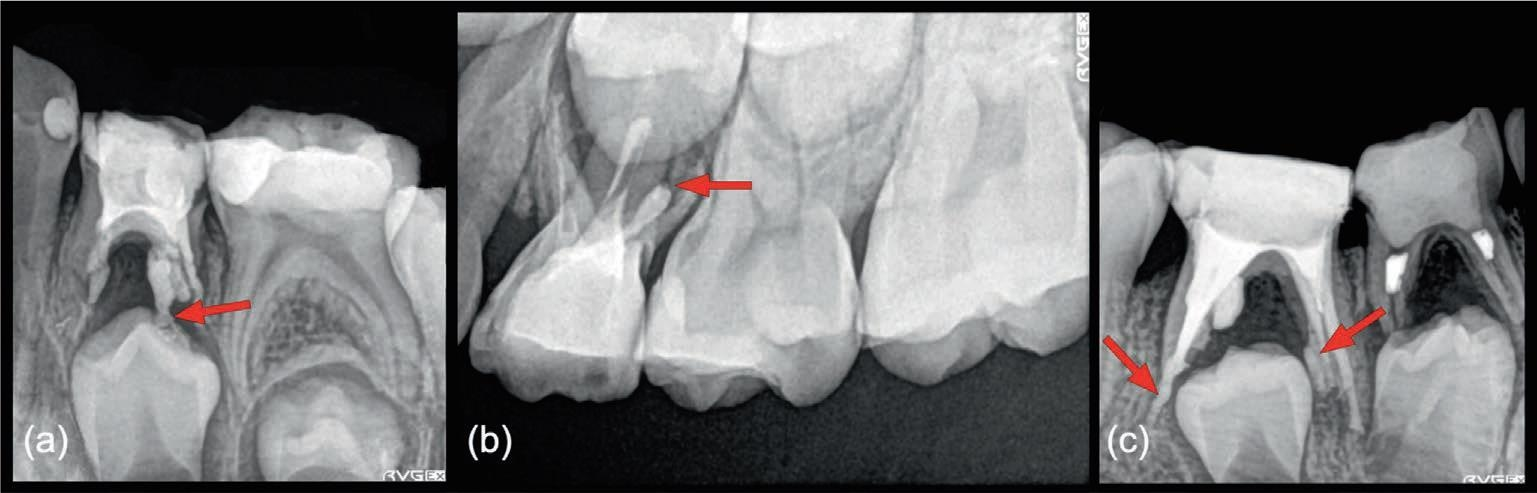
Examples of MTA extrusion directly after obturation. (a) (Case 7) Periapical radiograph of mandibular left first primary molar (No. L) after initial treatment showing furcation lesion, extrusion of compacted MTA expressed from both canals due to resorptive defects. Note the distal canal extruded MTA extending directly into the follicle and onto the occlusal surface of the permanent first premolar (arrow). (b) (Case 9) Radiograph of the left maxillary first primary molar (No. I) exhibiting voids in the mesio‐buccal and palatal roots. MTA extrusion and external inflammatory resorption are evident in the disto‐buccal root (arrow). (c) (Case 3) Periapical radiograph of the right and second mandibular primary molars (No. S,T). Both molars presented with previous pulpotomy treatments and demonstrated furcation pathosis. Postoperative image after MTA compaction No. T shows significant MTA overfill in the distal canal, extrusion into the distal furcation area and excess MTA in contact with the mesial aspect of the erupting second permanent premolar (arrows).

The outcomes from this limited case series suggests that MTA obturation may be an alternative treatment for primary teeth with advanced endodontic pathosis. Several features of MTA obturation that were examined in this case series and contributed to healing and continued tooth retention deserve consideration. Previous case reports have focused primarily on MTA extrusion during apexification procedures for immature permanent teeth, with limited data available for primary teeth. Although these reports recommended avoiding its occurrence, all cases with apical periodontitis and apical root resorption healed uneventfully without surgical intervention even when significant volumes of MTA were extruded in cases with periapical lesions [33, 34]. In the presence of open apices, furcation foramina and undetected resorptive defects perforating the canal walls or pulp chamber floor often make some degree of MTA extrusion unavoidable when obturation is performed without pretreatment barriers. (Figure 2). In most cases included in this study, the placement of barriers was not clinically feasible.

**Figure 3:**
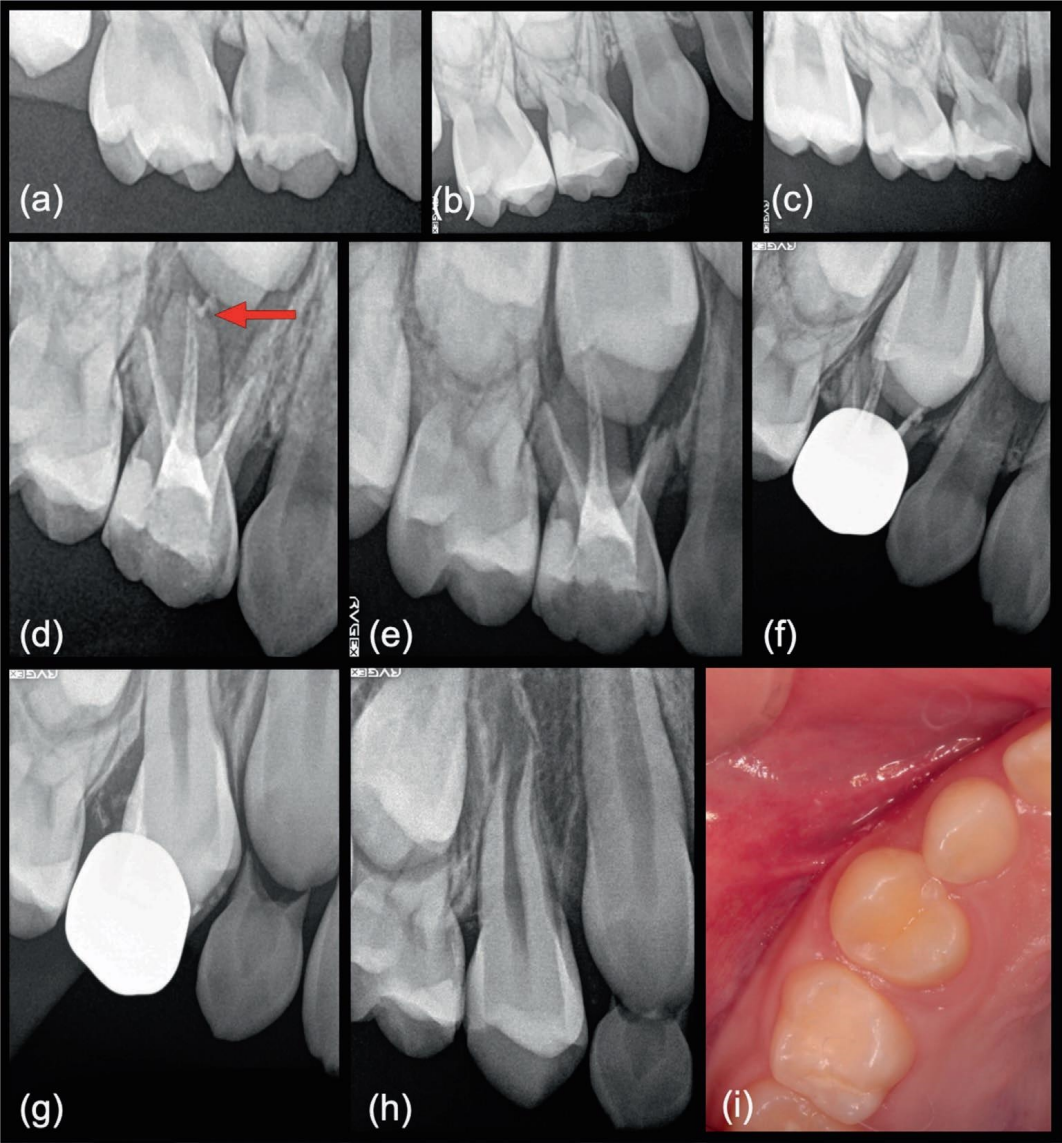
Association of primary tooth and successor tooth follicle (Case 19). (a) Bitewing radiograph revealing interproximal caries between maxillary right first and second molars (No. A,B) in an 8‐year 2‐month‐old female patient. (b) Periapical image showing completed restoration No. B after direct pulp capping using Endosequence BC Putty. (c) Two‐year recall radiograph showing furcation pathosis. The patient was symptomatic and diagnosed with pulpal necrosis. (d) Postoperative radiograph showing completed MTA obturation with bonded composite restoration. Note extrusion of MTA in close proximity with the tooth follicle of the maxillary permanent first premolar (arrow). (e) Four‐month radiographic review. (f) One‐year recall with zirconia crown showing normal primary tooth resorption and continued eruption of the permanent premolar. (g) Two‐year periapical radiograph prior to elective extraction. (h, i) Two‐year 6‐month radiographic review and intraoral photograph showing fully erupted unaffected premolar. Note absence of residual MTA in supporting bone or soft tissues.

Before the introduction of MTA and CSCs, PE in primary teeth with pulp chamber floor perforations, advanced resorptive defects, follicular involvement, or cystic pathology were generally contraindicated [1, 5]. Notwithstanding, many pretreatment cases in this study were characterized by these very features (Figure 3). It appears that in all cases, even when characterized by advanced loss of supporting bone and inflammatory root resorption, reformation/healing and stabilization of the periodontium was consistently observed when MTA was used as the filling material. These observations suggest that MTA may allow prolonged retention of infected primary teeth that previously would have been designated for extraction had conventional materials been employed [1, 2, 9].

**Figure 4:**
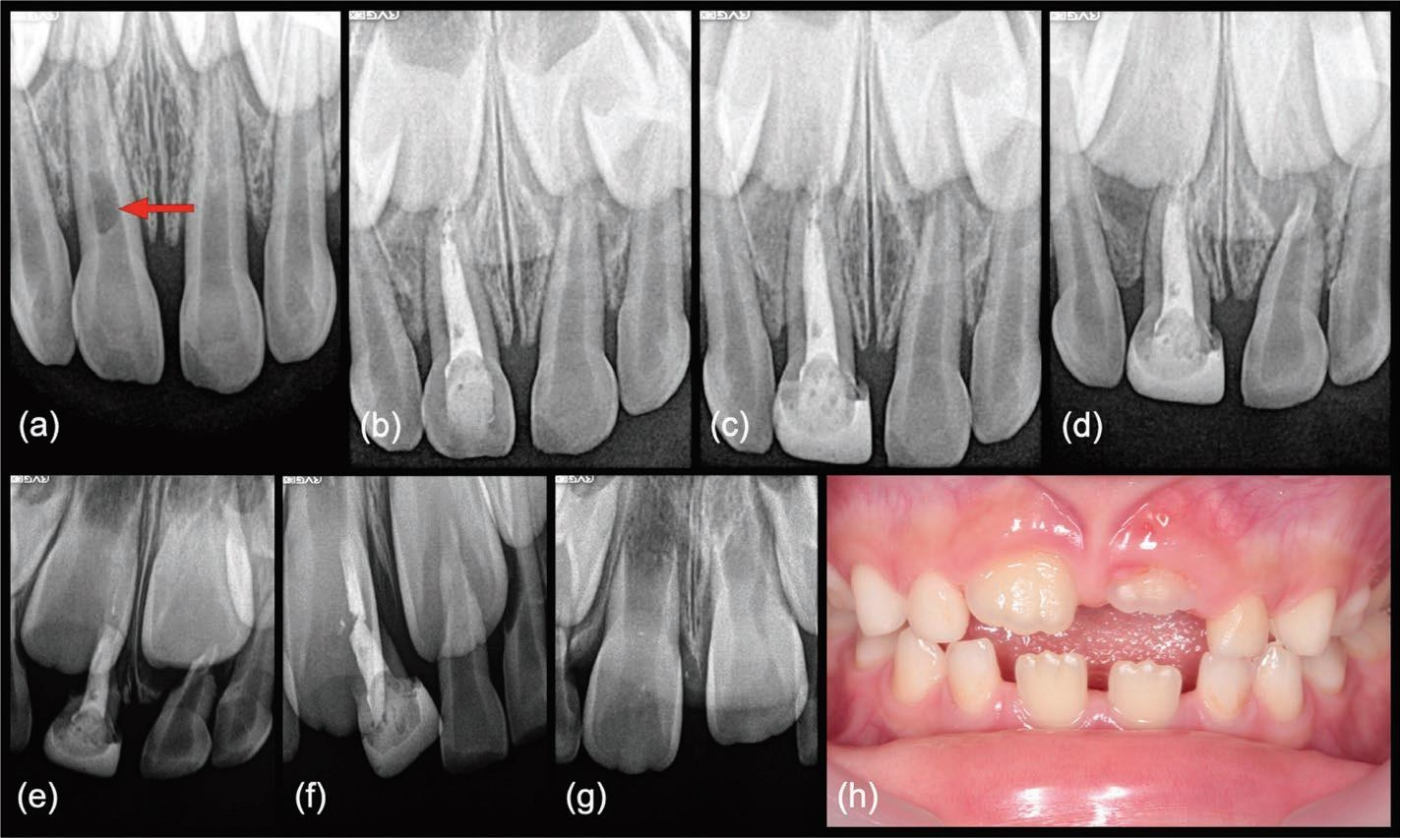
Elective extraction with residual MTA removal (Case 10). (a) Preoperative radiograph of a 3‐year 9‐month‐old symptomatic male patient presenting with history of trauma to the maxillary right primary central incisor (No. D) displaying external root resorption (arrow). (b) Post‐operative radiograph after root canal treatment and MTA obturation. (c) Four‐month post‐operative radiograph after placement of resin strip crown. (d) One‐year, 9‐month radiographic recall. Note arrest of ongoing external resorption. (e) Radiograph showing 2‐year, 10‐month follow‐up. (f) Three‐year, 7‐month radiograph prior to elective extraction. (g) Two‐week follow‐up radiograph after routine extraction and atraumatic curettage combined with water spray irrigation/flushing removal of remaining MTA. (h) Clinical photograph showing normal eruption pattern of the permanent central incisor.

**Figure 5:**
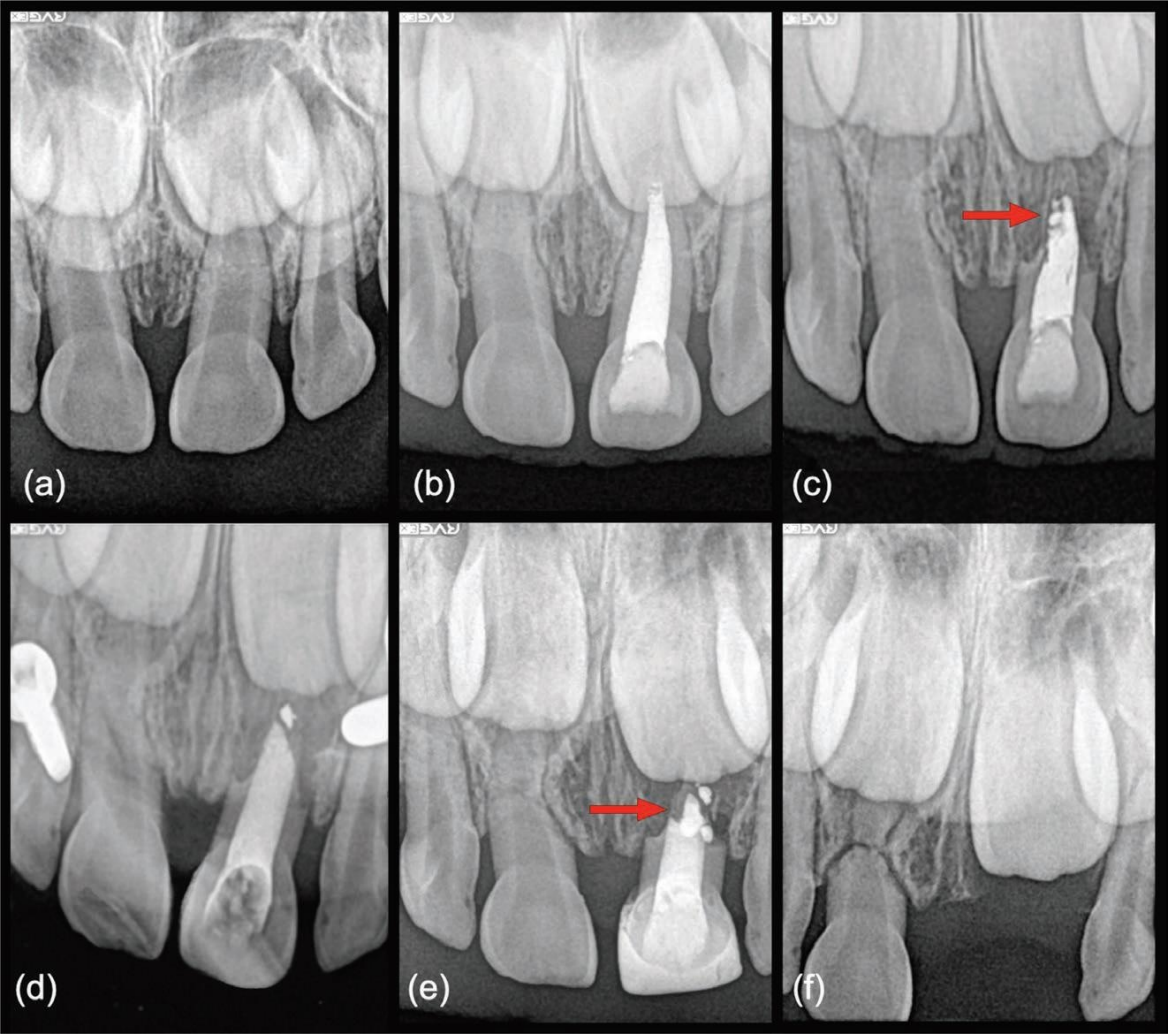
Retreatment using MTA obturation (Case 17). (a) Periapical radiograph of maxillary left primary left central incisor (No. F) associated with previous trauma in a 3‐year 10‐month‐old female patient. (b) Periapical radiograph after root canal obturation with Vitapex and access cavity bonded composite. (c) Two‐year recall radiograph after patient returned with swelling of the buccal plate and pain. Note continued apical root resorption, periapical bone loss and ongoing resorption of the obturation paste (arrow). Treatment included paste removal and PE retreatment with MTA root canal obturation. (d) One‐month recall radiograph. The patient exhibited resolution of swelling and symptomology. (e) One‐year follow‐up radiograph showing placement of a strip crown and continued root resorption and resorption/fragmentation of the MTA cement (arrow). The tooth was uneventfully extracted with soft tissue containing all residual MTA. (f) One‐year 6‐month follow‐up after MTA retreatment, showing normal eruption pattern of the succedaneous tooth without MTA retention in soft tissues or bone.

The application of MTA as an PE obturation material challenges the longstanding concept that root canal filling materials in primary teeth must resorb at a rate synchronized with physiologic root resorption. Although extruded MTA resorbs more slowly than the natural exfoliative process, all nonresorbed cement was ultimately eliminated without complication—either during exfoliation, elected extraction, or selective curettage combined with water irrigation/flushing (Figures 4 and 5). Notably, fewer than one‐quarter of cases (23.8%, *n* = 5) required active removal of residual MTA at the time of exfoliation or tooth removal (Table 2). These included three partially resorbed roots retained after molar exfoliation, one separated root after elected molar extraction, and one case managed with atraumatic curettage and water irrigation/flushing for removal of residual cement and unresorbed root fragments (Figure 4).

Another noteworthy consideration of this case series is the higher than normal extraction rate for initially PE treated or retreated teeth. This rate is not unexpected in pediatric practices in Tokyo, Japan, where access to pediatric specialists can be limited. In this setting, it is not uncommon practice for untreated or PE treated primary teeth to be intentionally extracted to prevent ectopic or delayed eruption after referral by general practitioners [35, 36]. In primary molars, it is not unusual for the eruption pattern of the succedaneous teeth to not be centrically aligned, leaving partially resorbed roots that obstruct normal eruption pathways [36]. Hence, intervention with planned extraction decreases the incidence of ectopic eruptions as reflected in this case series.

Assessments of certain case characteristics was limited without the benefit of cone‐beam computed tomography (CBCT) imaging. The identification of resorptive defects prior to MTA obturation using 2D radiographs can be challenging and often become evident only after MTA filling and extrusion. Furthermore, differentiating between true healing in the furcation area and bone changes associated with physiologic exfoliation was difficult. Consequently, outcomes were established as “stable” in the absence of re‐emergent disease.

An interesting secondary observation occurred in one patient who received PE for three molars. Remarkably, one contralateral tooth exfoliated 3 years and 7 months after the corresponding treated tooth. This outlier contributed to the longer than expected mean replacement period for the contralateral teeth (approximately 7 months), considered to be later than average based on untreated antimeres at the 6‐month recall period [9]. The delayed eruption may have been related to genetic factors or an undiagnosed endocrine condition.

In cases with advanced root resorption, it appears MTA can promote healing of the periodontium without adversely altering successor teeth replacement periods. No instances of MTA overfill or extrusion resulted in damage to the permanent tooth follicles. Additionally, residual extruded MTA was not detectable in bone after exfoliation or extraction, and when detected in soft tissues, it was removed atraumatically. Importantly, all fully erupted succedaneous teeth showed no evidence of enamel defects or hypoplasia. Although this investigation is limited by the sample size and absence of pre/post‐treatment CBCT imaging, observations from this case study may stimulate future randomized clinical trials or case‐controlled studies that can strengthen support for this treatment option. In addition, this case series may encourage the development of CSC‐based products specifically designed for primary teeth, with resorption rates more closely aligned with physiologic exfoliative resorption while preserving the beneficial biological and physicochemical properties. Considering the limitations of currently available obturation materials, MTA and future CSC‐based products may provide a more biologically oriented strategy for improving retention and outcomes in primary teeth with advanced pathosis.

## 5. Conclusion

The outcomes of this case series suggest that MTA cement may be an alternative PE root canal filling material option for initial treatment or retreatment in infected primary teeth. In cases with significant root resorption, MTA appears to promote healing of the periodontium without adversely altering successor teeth replacement periods. Additionally, residual extruded MTA is not detectable in bone after exfoliation or extraction. In cases where MTA was detected in soft tissues after exfoliation, it was atraumatically removed or shed during permanent tooth eruption. All succedaneous teeth in this limited case series exhibited normal enamel development, with no evidence of defects or hypoplasia after complete eruption.

## Author Contributions

**Tetsuhide Makiguchi:** conceptualization, investigation, methodology, resources, supervision, data curation, writing—original draft, and writing—review and editing. **Yoshi Terauchi:** data curation, investigation, and writing—review and editing. **Kingsley Wong:** data curation, formal analyses, investigation, software, and writing—review and editing. **George Bogen:** conceptualization, data curation and analyses, investigation, visualization, writing—original draft, and writing—review and editing.

## Funding

This study did not receive any specific grant from funding agencies in the public, commercial, or not‐for‐profit sectors. Open access publishing facilitated by The University of Queensland, as part of the Wiley ‐ The University of Queensland agreement via the Council of Australasian University Librarians.

## Consent

The authors certify that they have obtained all appropriate patient consent forms. In the form, the parents or guardians of patients have given consent for treatment, including patient images and other clinical information to be reported in the journal. The parents or guardians understand that patient names and initials will not be published and due efforts will be made to conceal their identity, but anonymity cannot be guaranteed.

## Conflicts of Interest

The authors declare no conflicts of interest.

## Endnotes

^1^Pediatric dentistry defines pulpectomy as a root canal procedure for primary teeth with irreversibly inflamed or necrotic pulp resulting from caries or trauma [2].

## Data Availability

The data that support the findings of this study are available from the corresponding author upon reasonable request.
